# FOXO3-engineered human mesenchymal stem cells efficiently enhance post-ischemic stroke functional rehabilitation

**DOI:** 10.1093/procel/pwaf004

**Published:** 2025-01-17

**Authors:** Fangshuo Zheng, Jinghui Lei, Zan He, Taixin Ning, Shuhui Sun, Yusheng Cai, Qian Zhao, Shuai Ma, Weiqi Zhang, Jing Qu, Guang-Hui Liu, Si Wang

**Affiliations:** Key Laboratory of Organ Regeneration and Reconstruction, State Key Laboratory of Membrane Biology, State Key Laboratory of Stem Cell and Reproductive Biology, Institute of Zoology, Chinese Academy of Sciences, Beijing 100101, China; Department of Neurology, The Fifth People’s Hospital of Chongqing, Chongqing 400062, China; Advanced Innovation Center for Human Brain Protection, National Clinical Research Center for Geriatric Disorders, Xuanwu Hospital, Capital Medical University, Beijing 100053, China; Aging Translational Medicine Center, Beijing Municipal Geriatric Medical Research Center, Xuanwu Hospital, Capital Medical University, Beijing 100053, China; Advanced Innovation Center for Human Brain Protection, National Clinical Research Center for Geriatric Disorders, Xuanwu Hospital, Capital Medical University, Beijing 100053, China; Aging Translational Medicine Center, Beijing Municipal Geriatric Medical Research Center, Xuanwu Hospital, Capital Medical University, Beijing 100053, China; Advanced Innovation Center for Human Brain Protection, National Clinical Research Center for Geriatric Disorders, Xuanwu Hospital, Capital Medical University, Beijing 100053, China; Aging Translational Medicine Center, Beijing Municipal Geriatric Medical Research Center, Xuanwu Hospital, Capital Medical University, Beijing 100053, China; Beijing Institute of Heart Lung and Blood Vessel Diseases, Beijing Anzhen Hospital, Capital Medical University, Beijing 100029, China; Key Laboratory of Organ Regeneration and Reconstruction, State Key Laboratory of Membrane Biology, State Key Laboratory of Stem Cell and Reproductive Biology, Institute of Zoology, Chinese Academy of Sciences, Beijing 100101, China; Beijing Institute for Stem Cell and Regenerative Medicine, Beijing 100101, China; Advanced Innovation Center for Human Brain Protection, National Clinical Research Center for Geriatric Disorders, Xuanwu Hospital, Capital Medical University, Beijing 100053, China; Aging Translational Medicine Center, Beijing Municipal Geriatric Medical Research Center, Xuanwu Hospital, Capital Medical University, Beijing 100053, China; Key Laboratory of Organ Regeneration and Reconstruction, State Key Laboratory of Membrane Biology, State Key Laboratory of Stem Cell and Reproductive Biology, Institute of Zoology, Chinese Academy of Sciences, Beijing 100101, China; Beijing Institute for Stem Cell and Regenerative Medicine, Beijing 100101, China; Aging Biomarker Consortium, Beijing 100101, China; Aging Biomarker Consortium, Beijing 100101, China; University of Chinese Academy of Sciences, Beijing 100049, China; CAS Key Laboratory of Genomic and Precision Medicine, Beijing Institute of Genomics, Chinese Academy of Sciences, Beijing 100101, China; China National Center for Bioinformation, Beijing 100101, China; Sino-Danish College, University of Chinese Academy of Sciences, Beijing 101408, China; Sino-Danish Center for Education and Research, Beijing 101408, China; Key Laboratory of Organ Regeneration and Reconstruction, State Key Laboratory of Membrane Biology, State Key Laboratory of Stem Cell and Reproductive Biology, Institute of Zoology, Chinese Academy of Sciences, Beijing 100101, China; Beijing Institute of Heart Lung and Blood Vessel Diseases, Beijing Anzhen Hospital, Capital Medical University, Beijing 100029, China; Beijing Institute for Stem Cell and Regenerative Medicine, Beijing 100101, China; Aging Biomarker Consortium, Beijing 100101, China; University of Chinese Academy of Sciences, Beijing 100049, China; Key Laboratory of Organ Regeneration and Reconstruction, State Key Laboratory of Membrane Biology, State Key Laboratory of Stem Cell and Reproductive Biology, Institute of Zoology, Chinese Academy of Sciences, Beijing 100101, China; Advanced Innovation Center for Human Brain Protection, National Clinical Research Center for Geriatric Disorders, Xuanwu Hospital, Capital Medical University, Beijing 100053, China; Beijing Institute for Stem Cell and Regenerative Medicine, Beijing 100101, China; Aging Biomarker Consortium, Beijing 100101, China; University of Chinese Academy of Sciences, Beijing 100049, China; Advanced Innovation Center for Human Brain Protection, National Clinical Research Center for Geriatric Disorders, Xuanwu Hospital, Capital Medical University, Beijing 100053, China; Aging Translational Medicine Center, Beijing Municipal Geriatric Medical Research Center, Xuanwu Hospital, Capital Medical University, Beijing 100053, China; Aging Biomarker Consortium, Beijing 100101, China

**Dear Editor,**

Ischemic stroke, a well-known age-related disorder (Cai et al., 2022b), resulting from the occlusion of cerebral blood vessels and the ensuing neuronal damage, has emerged as a leading cause of mortality and disability worldwide (Zhang and Chopp, 2009). It represents a significant global health challenge. The pursuit of innovative treatment strategies for ischemic stroke has become an urgent scientific priority. Stem cell therapy has gained prominence as a promising therapeutic modality for attenuating ischemic brain injury and facilitating repair in affected regions (Zhu et al., 2023). Among these, mesenchymal stem cells (MSCs) have garnered particular attention for their potential in ischemic stroke therapy, attributed to their capacity to secrete therapeutic biomolecules that provide neuroprotection, stimulate angiogenesis, and modulate immune responses (Stonesifer et al., 2017). Despite their promise, a multitude of challenges impede the realization of MSCs’ therapeutic potential. For instance, the variability in the sources of primary MSCs presents challenges in reliably obtaining a sufficient quantity of cells for transplantation. Additionally, as the number of passages increases, MSCs exhibit increased cellular senescence and a diminished capacity for differentiation. Moreover, the harsh microenvironment within the recipient ischemic tissue often leads to inadequate retention and survival of transplanted cells at the target site (Cai et al., 2022a; Stonesifer et al., 2017). Consequently, developing strategies to provide superior cell materials is essential for enhancing the therapeutic efficacy of MSC-based treatments. Recent studies have shown that targeted gene editing in stem cells can boost their functionality, leading to improved post-transplant survival and therapeutic benefits (Cai et al., 2022a). Our previous studies have highlighted that activating *Forkhead box O3* (*FOXO3)*, a gene linked to longevity, in MSCs derived from human embryonic stem cells (hESCs) reduces cellular senescence, boosts self-renewal, and strengthens stress resistance (Lei et al., 2021; Yan et al., 2019).

In our study, we demonstrated that intracerebral implantation of FOXO3-engineered human mesenchymal stem cells (F3-MSCs) advances functional recovery in mice after ischemic stroke induced by middle-cerebral-artery-occlusion (MCAO). The therapeutic efficacy of MSCs was primarily attributed to their paracrine signaling. Indeed, exosomes secreted by F3-MSCs demonstrated a comparable ability to modulate the microenvironment and promote the recovery at the lesion region, indicating that these extracellular vesicles play an important role in mediating the beneficial effects of MSC therapy. By collectively mitigating post-stroke inflammation, regulating scar tissue formation, stimulating neovascularization, and enhancing neurogenesis, this intervention represents an advancement in the treatment of ischemic stroke.

Initially, to evaluate the therapeutic potential of F3-MSCs in ischemic stroke, we developed a permanent unilateral MCAO mouse model (Fig. 1A). This model emulates the pathophysiological conditions of human ischemic stroke and is widely employed in preclinical research for its fidelity to the human condition (Iadecola and Anrather, 2011; Stonesifer et al., 2017). Consistently, our observation revealed a reduction in cerebral blood flow on the operated side of the MCAO mice monitored by a laser speckle flowmetry when contrasted with the sham-operated group (Fig. 1B). It is well-documented that the motor and sensory functions serve as indicators of their neurological status, and these can be assessed through the cylinder test and the adhesive removal test, respectively. In the behavioral assessments, we discerned that the mice exhibited impaired coordination in their limb movements and diminished sensory capabilities on the side contralateral to the insult (Fig. 1C and 1D).

**Figure 1.**
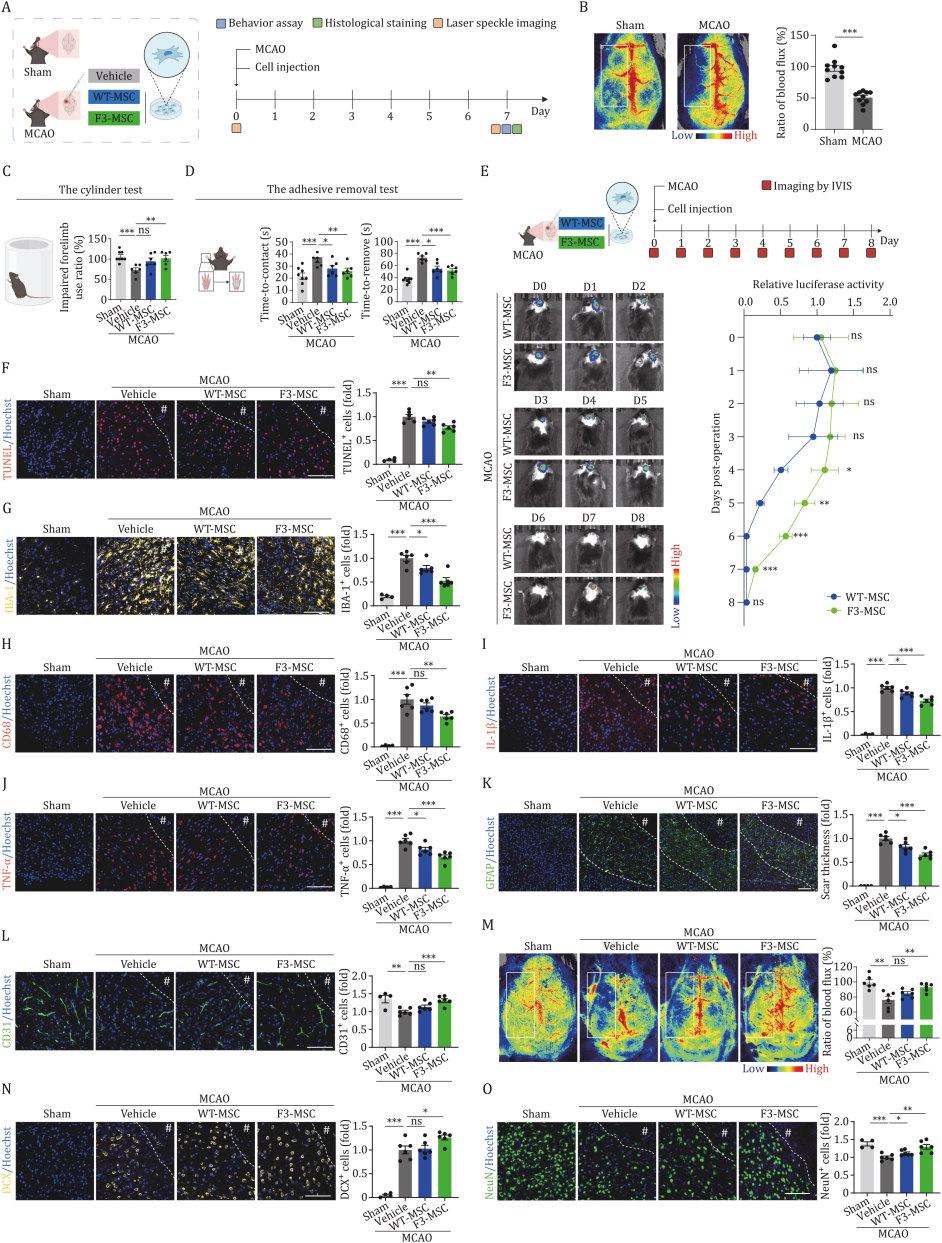
Intracerebral implantation of F3-MSCs improves cell engraftment and promotes post-stroke functional recovery. (A) Schematic diagram of experimental design for MSC implantation and phenotypical analysis. (B) Representative cerebral blood flow images of sham group and MCAO mice. Laser speckle flowmetry was measured immediately post-stroke. Ratios of blood flux were quantified and are presented as mean ± SEMs. *n* = 10 mice for each group. (C) Behavior analysis by the cylinder test at D7 post-stroke. Impaired forelimb use ratios (impaired forelimb/unimpaired forelimb) were quantified and are shown as the mean ± SEMs. *n *= 8 for sham group; *n* = 6 for the other groups. (D) Behavior analysis by the adhesive removal test at D7 post-stroke. The contact time (left) and removal time (right) were quantified and are shown as the mean ± SEMs. *n* = 8 for sham group; *n* = 6 for the other groups. (E) MSC retention evaluation using *in vivo* bioluminescence imaging of luciferase-labeled MSCs. C57/B6J mice with MCAO were subjected to intracranial injection of luciferase-labeled WT-MSCs or F3-MSCs. Luciferase activity was detected and quantified using an *in vivo* imaging system. Quantitative data of luciferase activity are presented as the mean ± SEMs. *n* = 8 for each group. (F) Analysis of TUNEL-positive apoptotic cells in peri-infarct border zone of mouse brain at D7 post-stroke. Left, representative fluorescence images. Right, TUNEL-positive cells were quantified as fold changes, which were normalized to that of the vehicle group. Data are presented as the mean ± SEMs. *n* = 4 for sham group; *n* = 6 for the other groups. Scale bar, 100 μm. The white dotted line denotes the lesion border after ischemic stroke; # indicates the stroke lesion region. (G) Analysis of IBA-1-positive microglia in peri-infarct border zone of mouse brain at D7 post-stroke. Left, representative fluorescence images. Right, IBA-1-positive cells were quantified as fold changes, which were normalized to that of the vehicle group. Data are presented as the mean ± SEMs. *n* = 4 for sham group; *n* = 6 for the other groups. Scale bar, 100 μm. The white dotted line denotes the lesion border after ischemic stroke; # indicates the stroke lesion region. (H) Analysis of CD68-positive activated microglia in peri-infarct border zone of mouse brain at D7 post-stroke. Left, representative fluorescence images. Right, CD68-positive cells were quantified as fold changes, which were normalized to that of the vehicle group. Data are presented as the mean ± SEMs. *n* = 4 for sham group; *n* = 6 for the other groups. Scale bar, 100 μm. The white dotted line denotes the lesion border after ischemic stroke; # indicates the stroke lesion region. (I and J) Analysis of the expression of IL-1β (Ι) and TNF-α (J) in peri-infarct border zone of mouse brain at D7 post-stroke. Left, representative fluorescence images. Right, IL-1β-positive (Ι) and TNF-α-positive (J) cells were quantified as fold changes, which were normalized to that of the vehicle group. Data are presented as the mean ± SEMs. *n* = 4 for sham group; *n* = 6 for the other groups. Scale bar, 100 μm. The white dotted line denotes the lesion border after ischemic stroke; # indicates the stroke lesion region. (K) Analysis of GFAP-positive astrocytes in peri-infarct border zone of mouse brain at D7 post-stroke. Left, representative fluorescence images. Right, the thickness of astrocytic scars was quantified as fold changes, which were normalized to that of the vehicle group. Data are presented as the mean ± SEMs. *n* = 4 for sham group; *n* = 6 for the other groups. Scale bar, 100 μm. The white dotted lines denote the border of the scar after ischemic stroke; # indicates the stroke lesion region. (L) Analysis of the CD31-positive endothelial cells in peri-infarct border zone of mouse brain at D7 post-stroke. Left, representative fluorescence images. Right, CD31-positive cells were quantified as fold changes, which were normalized to that of the vehicle group. Data were quantified and are presented as the mean ± SEMs. *n* = 4 for sham group; *n* = 6 for the other groups. Scale bar, 100 μm. The white dotted line denotes the lesion border after ischemic stroke; # indicates the stroke lesion region. (M) Representative cerebral blood flow images of mice in sham group, as well as MCAO mice injected with vehicle, WT-MSCs and F3-MSCs. Laser speckle flowmetry was measured at D7 after operation. Ratios of blood flux were quantified and are presented as mean ± SEMs. *n* = 6 mice per group. (N) Analysis of the DCX-positive cells in peri-infarct border zone of mouse brain at D7 post-stroke. Left, representative fluorescence images. Right, DCX-positive cells were quantified as fold changes, which were normalized to that of the vehicle group. Data are presented as the mean ± SEMs. *n* = 4 for sham group; *n* = 6 for the other groups. Scale bar, 100 μm. The white dotted line denotes the lesion border after ischemic stroke; # indicates the stroke lesion region. (O) Analysis of the NeuN-positive mature neurons in peri-infarct border zone of mouse brain at D7 post-stroke. Left, representative fluorescence images. Right, NeuN-positive cells were quantified as fold changes, which were normalized to that of the vehicle group. Data are presented as the mean ± SEMs. *n* = 4 for sham group; *n *= 6 for the other groups. Scale bar, 100 μm. The white dotted line denotes the lesion border after ischemic stroke; # indicates the stroke lesion region. *, *P* < 0.05; **, *P* < 0.01; ***, *P* < 0.001; ns, not significant (two-tailed Student’s *t* test).

Next, we derived wild-type (WT)-MSCs and F3-MSCs through directed differentiation of WT-hESCs and *FOXO3-*genetically modified hESCs. The genetic modification was achieved by utilizing gene editing technique to substitute two critical FOXO3 phosphorylation sites with alanine residues (S253A and S315A) (Fig. S1A and S1B), as previously described (Lei et al., 2021; Yan et al., 2019). The genetically modified FOXO3 was rendered refractory to AKT-mediated phosphorylation at the S253 and S315 sites, resulting in its sustained nuclear activity. In alignment with the forementioned studies (Lei et al., 2021; Yan et al., 2019), our F3-MSCs demonstrated enhanced self-renewal capacity and alleviated senescence compared to their wild-type counterparts (WT-MSCs) (Fig. S1C–E). Next, we aimed to quantify the *in vivo* retention capabilities of F3-MSCs subsequently to intracerebral implantation immediately after ischemic stroke caused by MCAO (Fig. 1A). We introduced a lentiviral construct expressing luciferase into both WT-MSCs and F3-MSCs before implanting them into MCAO mouse models (Fig. 1E). The bioluminescence, serving as a proxy for cell retention, was monitored using an *in vivo* imaging system (Fig. 1E). At the initial timepoint post-transplantation, the bioluminescent signals were observed to be comparable in animals that received either F3-MSCs or WT-MSCs, indicating similar initial engraftment (Fig. 1E). As the observation period extended, the F3-MSCs group demonstrated a more pronounced luminescent signal, suggesting an enhanced retention profile over the WT-MSCs group (Fig. 1E). The luminescence of the WT-MSCs group gradually diminished, becoming virtually undetectable by day six (D6) following transplantation (Fig. 1E). In contrast, the luminescent signal from the F3-MSCs group remained above the threshold of detection up to D8 post-transplantation, thereby highlighting the superior retention of F3-MSCs (Fig. 1E). These observations indicate the heightened retention and survival of F3-MSCs within the ischemic brain, reinforcing their potential as a therapeutic intervention for ischemic stroke.

Subsequently, we explored the capacity of F3-MSCs to enhance neurological recovery at D7 post-stroke (Fig. 1A). Our data initially revealed that F3-MSCs enhance limb motor control function post-stroke, outperforming the vehicle control group (Fig. 1C). Additionally, cell therapy with both WT-MSCs and F3-MSCs ameliorated the impairments in limb sensory function induced by MCAO at D7 post-surgery, with F3-MSCs demonstrating a more pronounced therapeutic effect (Fig. 1D). Further, we endeavored to ascertain whether the implantation of F3-MSCs could provide neuroprotection against ischemic stroke. Consistent with our hypothesis, we observed a reduction in MCAO-induced cellular apoptosis, as evidenced by TUNEL staining in the mouse brain at D7 following MSC implantation (Fig. 1F). Notably, the F3-MSCs group exhibited a diminished level of apoptosis compared to the vehicle group (Fig. 1F). These findings suggest that F3-MSCs possess the capacity to attenuate apoptosis in the brain of MCAO mice, which may promote the recovery of neurological function following ischemic stroke.

The activation of local microglia and the secretion of inflammatory cytokines are recognized as principal inflammatory responses correlated with brain injury following a stroke (Iadecola and Anrather, 2011; Stonesifer et al., 2017). Given the potent immunomodulatory attributes of MSCs, we aimed to assess the impact of F3-MSCs treatment on local inflammatory processes. At one-week post-stroke induction, the transplantation of WT-MSCs and F3-MSCs resulted in a reduction in IBA-1-positive microglia counts within the peri-infarct border-zone, compared to the vehicle treatment group (Fig. 1G). CD68, a marker for macrophage lineage cells (Ma et al., 2024), is predominantly expressed in microglia associated with brain parenchyma. It labels lysosomes and is recognized as an indicator of activated phagocytic microglia (Sun et al., 2023). Accordingly, we found that the F3-MSCs-treated group showed a reduction in the accumulation of activated microglia caused by MCAO compared to the vehicle group at the D7 post-stroke, as indicated by a decrease in the number of CD68-positive reactive cells (Fig. 1H). Furthermore, the expression levels of pro-inflammatory cytokines, including tumor necrosis factor-alpha (TNF-α) and interleukin-1 beta (IL-1β), were elevated following MCAO (Fig. 1I and 1J). Contrasting the vehicle group, MSC implantation lowered the expression of pro-inflammatory factors, and F3-MSC treatment outperformed in mitigating inflammation (Fig. 1I and 1J).

Astrocytes represent the most populous glial cell type within the central nervous system (CNS), fulfilling pivotal roles in maintaining CNS homeostasis, providing nutritional support, and offering protection, thereby playing a crucial part in the physiological and pathological mechanisms of numerous nervous system disorders (Aging Biomarker Consortium et al., 2023; Tan et al., 2023). In the aftermath of stroke injury, astrocytes become activated, serving a protective function by circumscribing the injured, infarcted region during the initial phase (Zhang and Chopp, 2009). However, it is important to recognize that astrocytes, key players in both reactive gliosis and glial scar formation, have the capacity to suppress axonal elongation and impede neuronal regeneration (Rust et al., 2024). Indeed, upon assessing post-stroke glial scar formation following the transplantation of MSCs, as indicated by glial fibrillary acidic protein (GFAP) immunostaining, we observed a reduced area of glial scarring in the MSCs-implanted group compared to the vehicle group at D7 post-operation, with the F3-MSC group showing a superior effect (Fig. 1K). This finding suggests a potential regulatory effect of F3-MSCs on reactive gliosis and the associated glial scar formation.

In addition to mitigating a range of injuries induced by stroke, the implantation of MSCs can also augment the intrinsic regenerative capacity of the affected tissue. Notably, neovascularization post-stroke is recognized as a crucial factor closely linked to the prognosis of stroke patients (Rust et al., 2024; Zhang and Chopp, 2009). To assess this, we examined post-stroke neovascularization following the implantation of MSCs through CD31 immunostaining. As anticipated, animals that received F3-MSCs exhibited an enhanced CD31-positive cells surrounding the lesion site at D7 post-stroke induction, in contrast to those treated with vehicle (Fig. 1L). In corroboration, the implantation of F3-MSCs also led to an elevated restoration of cerebral blood flow which had been compromised by MCAO compared to the vehicle group, as measured by laser speckle flowmetry (Fig. 1M). Collectively, these findings indicate that F3-MSC transplantation promotes angiogenesis, thereby underscoring the therapeutic potential of these cells in enhancing post-stroke recovery.

On another front, it is well established that in the wake of brain injury, neural stem cells and their progeny neuroblasts within the subventricular zone undergo escalated proliferation, followed by migration toward the site of injury, and ultimately differentiate into functional neurons (Rust et al., 2024; Zhang and Chopp, 2009). With this in mind, we proceeded to explore the potential of F3-MSCs transplantation to augment the endogenous reparative capacity and stimulate neurogenesis in the post-stroke context. This was accomplished by assessing the expression of doublecortin (DCX), a marker indicative of neural precursors (Yang et al., 2024), and neuronal nuclei (NeuN), a marker for mature neurons, through immunostaining techniques (Fig. 1N and 1O). Our findings revealed an increment in the count of DCX-positive cells in the peri-infarct border zone of animals that underwent F3-MSCs implantation, as compared to those administered vehicle at D7 post-operation (Fig. 1N). Additionally, an increased proportion of NeuN-positive neurons was observed following MSCs implantation, with the F3-MSCs treatment group demonstrating a pronounced increase (Fig. 1O). These results collectively suggest an enhancement of neurogenesis within a murine model of ischemic stroke following F3-MSCs transplantation.

The findings presented collectively suggest that the administration of F3-MSCs holds promise for their multifaceted therapeutic potential in facilitating post-stroke recovery. It is acknowledged that the beneficial impact of transplanted MSCs in ischemic stroke is primarily due to their paracrine-mediated actions or bystander effects, which are induced by a wide array of secreted bioactive molecules, rather than direct cell replacement within the infarcted regions (Hermann et al., 2024; Stonesifer et al., 2017). Accordingly, we proceeded to assess the capacity of exosomes derived from F3-MSCs to foster brain regeneration in the same mouse model of ischemic stroke via paracrine mechanisms (Fig. 2A). Consistently, exosomes originating from F3-MSCs, referred to as F3-Exo (Fig. S2A), enhanced the recovery of motor and sensory functions, as evidenced by improved limb use in the cylinder test and the adhesive removal test conducted at D7 post-MCAO, compared to those in vehicle group (Fig. 2B and 2C). Concurrently, a reduction in the number of TUNEL-positive apoptotic cells was observed in the MCAO mice treated with F3-Exo (Fig. 2D). This discovery implies that F3-Exo may ameliorate the effects of ischemic stroke by diminishing cell death within the impacted areas of the brain. Our study further confirmed the therapeutic efficacy of F3-Exo in mitigating post-stroke inflammation and glial scarring, while simultaneously promoting angiogenesis and neurogenesis (Fig. 2E–M). Specifically, F3-Exo treatment resulted in a reduction of activated microglia, as indicated by the decreased positivity for IBA-1 and CD68 (Fig. 2E and 2F), along with diminished levels of pro-inflammatory cytokines TNF-α and IL-1β in the peri-lesional region at D7 post-MCAO (Fig. 2G and 2H), outperforming both the vehicle and exosomes originating from WT-MSCs (WT-Exo) groups. Furthermore, F3-Exo mitigated glial scar formation in MCAO mice brains, evidenced by a reduction in the thickness of GFAP-labeled astrocyte glial scar at D7 post-MCAO when compared to vehicle-treated mice (Fig. 2I). Additionally, treatment with F3-Exo led to an increase in CD31-positive endothelial cells and improved cerebral blood flow, as measured by immunostaining and laser speckle imaging (Fig. 2J and 2K). It also led to increased numbers of DCX-positive neuroblasts and NeuN-positive neurons in the peri-infarct border zone at D7 post-stroke (Fig. 2L and 2M), compared the effects of vehicle treatment.

**Figure 2.**
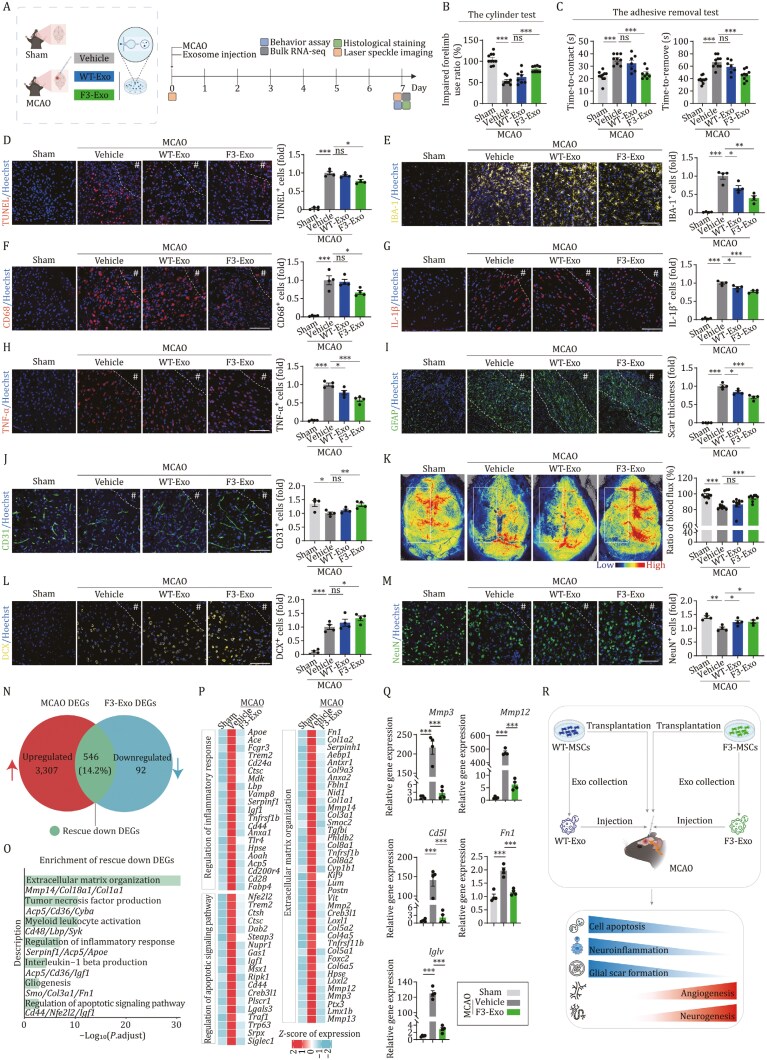
Intracerebral administration of F3-Exo promotes post-stroke functional recovery. (A) Schematic diagram of experimental design for exosome injection, phenotypical, and transcriptomic analysis. (B) The cylinder test at D7 post-stroke. Impaired forelimb use ratios (impaired forelimb/unimpaired forelimb) were quantified and are shown as the mean ± SEMs. *n* = 10 for sham group; *n* = 8 for the other groups. (C) The adhesive removal test at D7 post-stroke. The contact time (left) and removal time (right) were quantified and are shown as the mean ± SEMs. *n* = 10 for sham group; *n* = 8 for the other groups. (D) Analysis of TUNEL-positive apoptotic cells in peri-infarct border zone of mouse brain at D7 post-stroke. Left, representative fluorescence images. Right, TUNEL-positive cells were quantified as fold changes, which were normalized to that of the vehicle group. Data are presented as the mean ± SEMs. *n* = 4 for each group. Scale bar, 100 μm. The white dotted line denotes the lesion border after ischemic stroke; # indicates the stroke lesion region. (E) Analysis of IBA-1-positive microglia in peri-infarct border zone of mouse brain at D7 post-stroke. Left, representative fluorescence images. Right, IBA-1-positive cells are quantified as fold changes, which were normalized to that of the vehicle group. Data are presented as the mean ± SEMs. *n* = 4 for each group. Scale bar, 100 μm. The white dotted line denotes the lesion border after ischemic stroke; # indicates the stroke lesion region. (F) Analysis of CD68-positive activated microglia in peri-infarct border zone of mouse brain at D7 post-stroke. Left, representative fluorescence images. Right, CD68-positive cells were quantified as fold changes, which were normalized to that of the vehicle group. Data are presented as the mean ± SEMs. *n* = 4 for each group. Scale bar, 100 μm. The white dotted line denotes the lesion border after ischemic stroke; # indicates the stroke lesion region. (G and H) Analysis of the expression of IL-1β (G) and TNF-α (H) in peri-infarct border zone of mouse brain at D7 post-stroke. Left, representative fluorescence images. Right, IL-1β-positive (G) and TNF-α-positive (H) cells were quantified as fold changes, which were normalized to that of the vehicle group. Data are presented as the mean ± SEMs. *n* = 4 for each group. Scale bar, 100 μm. The white dotted line denotes the lesion border after ischemic stroke; # indicates the stroke lesion region. (I) Analysis of GFAP-positive astrocyte cell in peri-infarct border zone of mouse brain at D7 post-stroke. Left, representative fluorescence images. Right, the thickness of astrocytic scars was quantified as fold changes, which were normalized to that of the vehicle group. Data are presented as the mean ± SEMs. *n* = 4 for each group. Scale bar, 100 μm. The white dotted lines denote the border of the scar after ischemic stroke; # indicates the stroke lesion region. (J) Analysis of the CD31-positive endothelial cell in peri-infarct border zone of mouse brain at D7 post-stroke. Left, representative fluorescence images. Right, CD31-positive cells were quantified as fold changes, which were normalized to that of the vehicle group. Data are presented as the mean ± SEMs. *n* = 4 for each group. Scale bar, 100 μm. The white dotted line denotes the lesion border after ischemic stroke; # indicates the stroke lesion region. (K) Representative cerebral blood flow images of MCAO mice injected with vehicle, WT-MSCs and F3-MSCs and sham group. Laser speckle flowmetry was measured at D7 post-stroke. Ratios of blood flux were quantified and are presented as mean ± SEMs. *n* = 10 for sham group; *n* = 8 for the other group. (L) Analysis of the DCX-positive cells in peri-infarct border zone of mouse brain at D7 post-stroke. Left, representative fluorescence images. Right, DCX-positive cells were quantified as fold changes, which were normalized to that of the vehicle group. Data are presented as the mean ± SEMs. *n* = 4 for each group. Scale bar, 100 μm. The white dotted line denotes the lesion border after ischemic stroke; # indicates the stroke lesion region. (M) Analysis of the NeuN-positive mature neurons in peri-infarct border zone of mouse brain at D7 post-stroke. Left, representative fluorescence images. Right, NeuN-positive cells were quantified as fold changes, which were normalized to that of the vehicle group. Data are presented as the mean ± SEMs. *n* = 4 for each group. Scale bar, 100 μm. The white dotted line denotes the lesion border after ischemic stroke; # indicates the stroke lesion region. (N) Venn diagram showing the numbers of upregulated MCAO DEGs, the downregulated F3-Exo DEGs, and the overlapping rescue down DEGs. The proportion of rescue down DEGs was also indicated. (O) Representative Gene Ontology (GO) terms enriched in rescue down DEGs based on functional enrichment analysis (*P*.adjust < 0.05). The length of the bar represents the −log_10_ (*P*.adjust) value. (P) Heatmaps showing the scaled expression levels of genes related to three representative functional terms. (Q) The expression changes of indicated genes were verified by RT-qPCR analysis. *n* = 4 mice per group. Data are shown as the mean ± SEMs. (R) The schematic illustration showing the therapeutic effects of F3-MSCs and their derived exosomes in MCAO mice. *, *P* < 0.05; **, *P* < 0.01; ***, *P* < 0.001; ns, not significant (two-tailed Student’s *t* test).

To further elucidate the molecular changes within the ischemic lesion following the intervention, we collected corresponding brain tissue samples from groups that received either F3-Exo or vehicle treatment, as well as from the sham-operated group. Subsequently, we conducted bulk RNA sequencing analysis on these samples. This process allowed us to identify differentially expressed genes (DEGs) between the vehicle and sham groups, and between the F3-Exo-treated group and the vehicle group, which we termed “MCAO DEGs” and “F3-Exo DEGs”, respectively (Table S1; Fig. S2B–S2F). Consistent with the phenotypes associated with ischemic stroke, the upregulated MCAO DEGs were predominantly involved in inflammatory responses, apoptosis, and gliogenesis, whereas the downregulated genes were linked to dendrite development (Fig. S2D). Next, our transcriptomic analysis also indicated that F3-Exo treatment counteracted the inflammation, apoptosis, and gliogenesis induced by MCAO, while also fostering neuronal differentiation (Fig. S2D). Upon conducting an integrative comparative analysis of these DEGs, we discovered a subset of genes, which we named “rescue DEGs” that were partially restored by F3-Exo intervention (Fig. 2N). This gene set included 546 DEGs that were upregulated by MCAO and subsequently downregulated by F3-Exo administration, with functions related to inflammation, apoptosis, and extracellular matrix (ECM) organization, which is pivotal for gliogenesis (Fig. 2O). Among these genes, we observed that metalloproteinase family members, such as *Mmp3* and *Mmp13*, which are capable of degrading the ECM and are also biomarkers for reactive neurotoxic astrocytes, were downregulated by F3-Exo (Fig. 2P). Additionally, genes associated with fibrosis, including *Fn1*, and *Col1a1*, were also upregulated by MCAO but subsequently rescued by F3-Exo treatment (Fig. 2P). We further validated several of these gene expression changes using RT-qPCR (Fig. 2Q), substantiating the beneficial effects exacted by F3-Exo. Interestingly, 15 rescue up DEGs were identified that also contributed to alleviating the pathological phenotypes. For instance, Ackr2, known as an interceptor and chemokine-scavenging receptor, was downregulated following MCAO but was upregulated following F3-Exo administration (Fig. S2E). In summary, F3-Exo possesses the capacity to suppress the transcriptional expression of genes associated with apoptosis, inflammation, and gliogenesis, while simultaneously promoting neovascularization and neurogenesis in the affected tissues. This dual action is instrumental in mitigating the pathological phenotype of infarcted tissues, offering a promising therapeutic strategy for ischemic stroke recovery (Fig. 2R).

The therapeutic efficacy of MSCs is largely attributed to their ability to engage in paracrine signaling (Hermann et al., 2024; Stonesifer et al., 2017). MSCs secrete exosomes, which are nanoscale, membrane-encapsulated vesicles (30–100 nm) that facilitate intercellular communication by conveying a spectrum of intricate biological molecules, such as growth factors, soluble proteins, cytokines, mRNAs, and miRNAs. These exosomes serve as conduits for MSCs to deliver therapeutic molecules to target cells, thus engaging in gene regulation and therapeutic action (Hermann et al., 2024). In our study, we discovered that exosomes derived from F3-MSCs contribute to the therapeutic process by promoting functional recovery following MCAO. This finding aligns with the restorative impact observed with direct MSC transplantation, suggesting that the therapeutic mechanism of MSCs in ischemic stroke might be mediated by the release of their exosomes. However, some studies have indicated that variations in environmental conditions can influence the composition, biogenesis, and secretion of exosomes, thereby altering their biological functions (Hermann et al., 2024). As such, the precise proteins or nucleic acids contained in F3-Exo that are responsible for the therapeutic effects in ischemic stroke require further investigation to elucidate their specific contributions to the treatment process.

Furthermore, we have utilized the intracranial direct injection transplantation method, which offers superior therapeutic benefits. However, its limitations, including the inability to maintain a consistent drug concentration and the invasive nature of the procedure, have hindered its broader clinical adoption (Stonesifer et al., 2017). The intravenous injection approach presents a more feasible alternative for transplantation, yet it also introduces distinct challenges. Notably, it struggles with poor distribution specificity, often resulting in unintended accumulation in off-target organs such as the liver and spleen. Additionally, the low efficiency of crossing the blood-brain barrier to achieve effective concentration at the target lesion can necessitate higher graft injection doses, potentially leading to increased side effects. Therefore, refining clinical treatment protocols—by optimizing drug delivery methods, dosages, and administration timing—could boost the efficacy of MSC and exosome therapies. Further, it has been reported that stem cells and their exosomes also have potential for treating conditions beyond ischemic stroke, including Alzheimer’s disease, intracerebral hemorrhage, and traumatic brain injury (Hermann et al., 2024; Rust et al., 2024; Stonesifer et al., 2017). Therefore, FOXO3-engineered MSCs, along with their exosomes, may also emerge as a promising therapeutic strategy for these complications.

In conclusion, we discovered that the implantation of F3-MSCs yielded superior therapeutic outcomes compared to WT-MSCs in mice afflicted with ischemic stroke. Initially, we noted that the intracranial transplantation of F3-MSCs enhanced early cell engraftment. The application of F3-MSCs and their exosomes amplified the efficacy of treatment for cerebral infarction, characterized by improvements in behavioral performance, a decrease in cell apoptosis, a reduction in inflammation and glial scar formation, the facilitation of angiogenesis, and the stimulation of neurogenesis. Our research suggests that FOXO3-engineered MSCs and their corresponding exosomes have a promising potential to be developed into therapeutic strategies for treating ischemic stroke.

## Supplementary data

Supplementary data is available at *Protein & Cell* online https://doi.org/10.1093/procel/pwaf004.

pwaf004_suppl_Supplementary_Material

## Footnotes

The authors are grateful to Jingyi Jia and Feifei Liu for the management of laboratory animals, Lei Bai, Qun Chu, Jing Lu, Ying Yang, Ruijun Bai, Luyang Tian, Xiuping Li and Xiangmei Jin for administrative assistance, Yihui Xu (IBP, CAS) for her optical *in vivo* tracing, and Lijuan Wang (IOZ, CAS) for the laser speckle flowmetry. RNA-seq data was processed on the “Era” petascale supercomputer of the Computer Network Information Center of CAS. We thank BioRender for providing icons and templates for drawing scheme in this study. This work was supported by the National Key R&D Program of China STI2030-Major Projects-2021ZD0202400, the National Natural Science Foundation of China (Grant Nos. 82125011, 81921006, 92149301, 92168201, 82122024), the National Key Research and Development Program of China (2020YFA0804000, 2022YFA1103700, 2020YFA0112200, 2022YFA1103802, 2021YFF1201000, 2023YFC3605400), the National Natural Science Foundation of China (82330044, 32341001, 92049304, 92049116, 32121001, 82192863, 82361148131, 82071588, 82361148130, 8231101626, 82201727, 82488301), CAS Project for Young Scientists in Basic Research (YSBR-076, YSBR-012), the Program of the Beijing Natural Science Foundation (Z230011, Z240018, JQ24044), the Informatization Plan of Chinese Academy of Sciences (CAS-WX2022SDC-XK14), New Cornerstone Science Foundation through the XPLORER PRIZE (2021-1045), Beijing Municipal Public Welfare Development and Reform Pilot Project for Medical Research Institutes (JYY2023-13), CAS Youth Interdisciplinary Team, Key Laboratory of Alzheimer’s Disease of Zhejiang Province (ZJAD-2024001), Excellent Young Talents Program of Capital Medical University (12300927), the Project for Technology Development of Beijing-affiliated Medical Research Institutes (11000023T000002036310), Excellent Young Talents Training Program for the Construction of Beijing Municipal University Teacher Team (BPHR202203105), and Beijing Hospitals Authority Youth Programme (QML20230806).

Fangshuo Zheng, Jinghui Lei, Zan He, Taixin Ning, Shuhui Sun, Yusheng Cai, Qian Zhao, Shuai Ma, Weiqi Zhang, Jing Qu, Guang-Hui Liu, and Si Wang declare that they have no conflict of interest. All institutional and national guidelines for the care and use of laboratory animals were followed. The authors declare no competing interests and their agreement to publish. All of them confirm their willingness to participate.

S.W., G.-H.L. and J.Q. conceptualized the work and supervised overall experiments. F.-S.Z. performed the phenotypic, mechanistic analyses and immunofluorescence staining. Z.H. performed bioinformatics analyses. J.-H.L. performed cell culture, exosomes collection and RT-qPCR. S.S., F.-S.Z., J.-H.L., Q.Z. and Y.-S.C. performed data analysis. J.Q., S.W., Z.H., G.-H.L. F.-S.Z. and J.-H.L. performed manuscript writing, review, and editing. All authors reviewed the manuscript.
