# Supplementary material for: FOXO3-engineered human mesenchymal stem cells efficiently enhance post-ischemic stroke functional rehabilitation
Source: Protein Cell. 2025 Jan 17;16(5):365–73. doi: 10.1093/procel/pwaf004 (PMC12120242; doi:10.1093/procel/pwaf004)

Materials and Methods

Generation and characterization of MSCs

Differentiation of hESCs into MSCs was carried out following established protocols (Yan et al., 2019). Briefly, embryoid bodies obtained by digestion of hESC were cultured in MSC differentiation medium, consisting of α-minimum essential medium (αMEM) supplemented with 10% fetal bovine serum (FBS), 10 ng/ml basic fibroblast growth factor (bFGF), 5 ng/ml transforming growth factor beta (TGF-β), and 1% penicillin/streptomycin, until the emergence of fibroblast-like cells. The cells were collected and purified with MSC-specific surface markers CD73, CD90, and CD105 and confirmed the absence of hematopoietic stem cell markers CD34, CD43, and CD45 by FACS. The primary antibodies used for FACS were as follows: anti-CD73 (Cat# 550257, BD), anti-CD90 (Cat# 555595, BD), anti-CD105 (Cat# 17-1057-41, eBioscience), anti-CD34 (Cat# 555822, BD), anti-CD43 (Cat# 560198, BD), anti-CD45 (Cat# 555482, BD).

Cell culture and exosome isolation

MSCs were cultured following previous study (Lei et al., 2021). Briefly, cells were cultured on 0.1% gelatin-coated plate with αMEM medium (GIBCO) supplemented with 10% FBS, 1% NEAA, 1% penicillin/streptomycin, 1% GlutaMAX and 1 ng/mL FGF-2 and incubated at 37°C with 5% CO_2. MSCs at passage 7 were collected for cell transplantation, and their culture medium was harvested for exosome isolation. To prevent exosome contamination from FBS, the FBS was subjected to an exosome

depletion process using ultracentrifugation at 100,000 g for a duration of 16 h (Lei et al., 2022). To collect exosomes, the conditioned medium was centrifuged at 500 g for 5 min and filtered with a 0.22 μm filter membrane to remove cell and cell debris. After ultracentrifuged with 100,000 g for 2 h at 4°C, the supernatants were removed and the exosomes were washed by 1 mL of saline, and recentrifuged at 100,000 g for an additional 2 h at 4ºC. To determine the concentration and size of the exosomes, nanoparticle tracking analysis (NTA) was employed.

Cell cycle phase analysis

MSCs were fixed in pre-cold 70% ethanol at -20°C overnight and washed with 1 x PBS twice. Subsequently, cells were resuspended in PBS supplemented with 0.2 mg/mL RNaseA, 0.02 mg/mL propidium iodide, and 0.1% Triton X-100, and incubated at 37°C for 30 min. Then, the stained samples were directly tested using BD FACS Aria II. Data were processed and analyzed using ModFit software.

Genomic DNA extraction and DNA sequencing

DNA sequencing was conducted to identify the specific edited sites within the *FOXO3* gene following gene editing. Genomic DNA was extracted using the DNeasy Blood & Tissue Kit protocol. Subsequently, PCR amplification was conducted with the PrimeSTAR DNA Polymerase Kit. The PCR products were sequenced at Beijing Tsingke Biotech Co., Ltd. The primers used in this study are listed in Table S2.

Clonal expansion assay

Clonal expansion assay was conducted as previously described (Lei et al., 2021). 3,000 MSCs were seeded in each well of a gelatin-coated 12-well plate and cultured for 10 days. Then, the cells were fixed and the cell density was calculated by ImageJ after crystal violet staining.

SA-β-gal staining

SA-β-gal staining was performed as previously described (Yan et al., 2019). In brief, MSCs were fixed in fixation buffer (2% formaldehyde and 0.2% glutaraldehyde) for 5 min at room temperature. After fixation, cells were washed for three times by PBS and then stained with staining solution at 37°C overnight. The percentage of SA-β-gal positive cells was quantified by ImageJ.

Animal experiments

All experimental procedures were conducted in compliance with the guide for the Care and Use of Laboratory Animals and adhered to the institutional animal ethics guidelines. These protocols were approved by the Institute of Zoology at the Chinese Academy of Sciences. All animals were fed under controlled 12-h light/dark conditions with free access to water and food.

Establishment of mouse ischemic stroke model and Laser speckle imaging

Male C57BL/6 mice, aged 8-12 weeks, were utilized to establish a model of

permanent distal middle cerebral artery occlusion (MCAO) via the electrocoagulation method (Llovera et al., 2014). Briefly, male C57BL/6 mice aged 8-12 weeks were anesthetized with saturated tribromoethanol. A 10 mm incision was made between the left ear and eye after disinfecting the surgical site. The temporal muscle was displaced, the skull was drilled, and the distal middle cerebral artery bifurcation was exposed. The proximal and distal segments were electrocoagulated using a coagulation pen to induce permanent distal MCAO. In the sham group, the procedure was limited to skull drilling, with no coagulation of the middle cerebral artery. Cerebral blood flow (CBF) changes were then measured using laser speckle flowmetry. Once the model was established, an incision was made under anesthesia to expose the entire skull. Subsequently, perfusion images were captured using the laser speckle imaging system (RWD Life Sciences, Beijing, CN). These images were recorded and analyzed to assess the changes in CBF ratio in the ischemic hemisphere relative to the uninjured contralateral hemisphere, with the latter serving as a normalization reference. The MCAO model is considered to be successfully established by a ~50% drop of baseline perfusion values (Ansari et al., 2011). In addition, the perfusion images of CBF were also collected and analyzed by the laser speckle system at the 7th day after operation.

Cell implantation and exosome injection

Immediately after the model was established, the laser speckle imaging system was first used to record the three edges of the infarct area on the cleaned skull. Subsequently, 6 μL of saline (as a vehicle) or 6 μL of saline containing either WT-

MSCs (6×10^5 cells) or F3-MSCs (6×10^5 cells) was administered intracranially to the three perilesional sites within the ischemic region. The injections were made at a depth of 1 mm from the skull surface using a gas-tight Hamilton syringe fitted with a 26-gauge needle (1×10^5 cells/μL, and 2 μL per injection site). For the exosome injection procedure, a solution of exosomes was prepared at a concentration of 100 μg/mL and administered in a 6 μL saline volume. This was then locally injected into specific brain regions as determined by laser speckle measurements, a method analogous to that used for MSC implantation. After that, the drilled hole was sealed with bone wax, the temporal muscle was recovered to the original position, and the wound was sutured.

***In vivo* bioluminescence imaging**

MSCs retention evaluation was performed by in vivo tracing of luciferase-labelled MSCs along with quantitative analysis (Lei et al., 2021). MSCs were transfected by lentiviral luciferase before delivered into the ischemic brain. The bioluminescence from transplanted MSCs were measured from 0 to 8 days after MCAO and cell administration by an IVIS Lumina XRMS Series III instrument.

The cylinder test

Mouse was placed into a vertical transparent glass cylinder (diameter: 8.8 cm; height: 17.5 cm) and an angle mirror was placed behind to observe the behavior of tested mouse. After adapted in the cylinder for 5 min, the numbers of paw touches including

left paw, right paw and both paws after full rear of a mouse were recorded. At least 20 contacts for one forelimb were recorded. After each test, the cylinder was cleaned with water and 70% ethanol to remove any odorant trace. The ratios of independent impaired forelimb use were calculated (Bouet et al., 2009).

The adhesive removal test

The adhesive removal test was performed according to previous study (Zaidi et al., 2021). Briefly, a small piece of adhesive tape (3 mm × 3 mm) was placed on the forepaw contralateral to the stroke hemisphere. Time taken to contact and remove adhesive tape was recorded, with a maximum of 120 s.

Histological analysis

The animals were anesthetized and subsequently euthanized via transcardiac perfusion with normal saline, followed by 4% paraformaldehyde (PFA). After the brains were harvested and fixed in 4% PFA overnight, they were cryoprotected in a 30% sucrose solution. Finally, the brains were coronally sectioned into 15-μm-thick slices using a cryostat microtome (CM1950, Leica, Wetzlar, Germany).

For immunofluorescence staining, 15-μm-thick brain sections were permeabilized with 0.4% Triton X-100 for 15 min at room temperature and then blocked with 5% BSA for 1 h. The slides were then incubated with primary antibodies against IBA-1 (Cat# 019-19741, Woke), GFAP (Cat# ab4674, Abcam), DCX (Cat# 4604S, Cell signaling Technology), NeuN (Cat# ab104224, Abcam), CD68 (Cat# ab125212,

Abcam), IL-1β (Cat# sc-52012, Santa Cruz), TNF-α (Cat# ab1793, Abcam) and CD31 (Cat# ab28364, Abcam) at 4°C overnight, labeled with fluorescent secondary antibodies at 37°C for 1 h, counterstained with Hoechst 33258 and mounted before being observed under a fluorescence microscope. Additionally, fluorescently labeled cells were manually counted in a blinded manner within three randomly selected fields in the peri-infarct area for each slide. To identify the apoptotic cells in brain, the terminal deoxy-nucleotidyl transferase-mediated d-UTP nick-end labeling (TUNEL) staining was performed by using an In Situ Cell Death Detection Kit (Roche, 11684795910), according to the manufacturer's instruction. The slides were scanned by laser scanning confocal microscope and analyzed using ImageJ software.

RNA analyses

Total RNA was extracted using TRIzol Reagent from mouse brain. cDNA was generated with the GoScript Reverse Transcription System (Promega). SYBR qPCR Mix (TOYOBO) was used in a CFX-384 Real-Time PCR system (Bio-Rad) to perform q-PCR. The GAPDH detected in the mouse brain was used as the internal control. Primers used in this study were listed in Table S2.

Bulk RNA-seq data processing

Fast-p (version 0.23.2) software was used for quality control, adapter trimming, quality filtering of raw bulk RNA-seq reads. HISAT2 (version 2.0.4) was then used for mapping the trimmed reads to the mm10 genome. The generated sam files were

then converted to bam files through SAMtools (version 1.6). The read count of each gene was calculated through the feature Counts (version 2.0.3) software. R package DESeq2 (version 1.2.4) was used to identify DEGs between samples (Vehicle/sham, F3-Exo/vehicle) with the cutoff values of adjusted P value < 0.05 and $|\text{Log}_2 \text{ FC}| \geq 0.5$.

Pathway enrichment analysis

Gene Ontology (GO) process and pathway enrichment analysis was performed by cluster-Profiler. Kappa-test scores were calculated between each two representative terms selected from the results (P value ≤ 0.05) and set as similarity score between terms.

Statistical analysis

Statistical analyses were performed using GraphPad Prism 8.0 software. Data are shown as the mean ± SEMs. Two-tailed Student's *t* test was used for comparing the difference between groups. Differences were considered statistically significant at P < 0.05.

Data availability

The raw sequencing data in this study has been deposited in the Genome Sequence Archive in the National Genomics Data Center, Beijing Institute of Genomics (China National Center for Bioinformation) of the Chinese Academy of Sciences, with accession number CRA017822.

References

Ansari, S., Azari, H., McConnell, D.J., Afzal, A., and Mocco, J. (2011). Intraluminal middle cerebral artery occlusion (MCAO) model for ischemic stroke with laser doppler flowmetry guidance in mice. J Vis Exp.

Bouet, V., Boulouard, M., Toutain, J., Divoux, D., Bernaudin, M., Schumann-Bard, P., and Freret, T. (2009). The adhesive removal test: a sensitive method to assess sensorimotor deficits in mice. Nat Protoc 4, 1560-1564.

Lei, J., Jiang, X., Li, W., Ren, J., Wang, D., Ji, Z., Wu, Z., Cheng, F., Cai, Y., Yu, Z.R., *et al.* (2022). Exosomes from antler stem cells alleviate mesenchymal stem cell senescence and osteoarthritis. Protein Cell 13, 220-226.

Lei, J., Wang, S., Kang, W., Chu, Q., Liu, Z., Sun, L., Ji, Y., Esteban, C.R., Yao, Y., Belmonte, J.C.I., *et al.* (2021). FOXO3-engineered human mesenchymal progenitor cells efficiently promote cardiac repair after myocardial infarction. Protein Cell 12, 145-151.

Llovera, G., Roth, S., Plesnila, N., Veltkamp, R., and Liesz, A. (2014). Modeling stroke in mice: permanent coagulation of the distal middle cerebral artery. J Vis Exp, e51729.

Yan, P., Li, Q., Wang, L., Lu, P., Suzuki, K., Liu, Z., Lei, J., Li, W., He, X., Wang, S., *et al.* (2019). FOXO3-Engineered Human ESC-Derived Vascular Cells Promote Vascular Protection and Regeneration. Cell Stem Cell 24, 447-461 e448.

Zaidi, S.K., Ahmed, F., Alkhatabi, H., Hoda, M.N., and Al-Qahtani, M. (2021). Nebulization of Low-Dose S-Nitrosoglutathione in Diabetic Stroke Enhances

Benefits of Reperfusion and Prevents Post-Thrombolysis Hemorrhage. Biomolecules 11.

Figure Legends

Figure S1. Characterization of F3-MSCs and their derived exosomes.

(A) Fluorescence-activated cell sorting (FACS) analysis of the expression of MSCs-specific markers (CD105, CD90, and CD73) and MSCs-irrelevant markers (CD45, CD43 and CD34).

(B) Representative images of WT-MSCs and F3-MSCs (left) and the schematic illustration of DNA sequencing analysis (right). Base conversion in the genomic DNA of F3-MSCs leads to the substitution of two serine residues at positions 253 and 315 with alanine in the FOXO3 protein. Scale bar, 100 μm. Red denotes the original amino acid or nucleotide in the WT-MSCs; blue denotes the edited ones in F3-MSCs.

(C) Cell cycle analysis of different cell cycle phases in WT-MSCs and F3-MSCs. Data are presented as mean ± SEMs, $n = 3$.

(D) Analysis of clonal expansion ability in WT-MSCs and F3-MSCs. Representative images of crystal violet staining (left) and the relative area of crystal violet-positive cells (right) using ImageJ software and shown as the mean ± SEMs, $n = 3$.

(E) SA-β-gal staining of WT-MSC and F3-MSC (passage 7). Scale bar, 100 μm. SA-β-gal-positive cells were quantified as fold changes (F3-MSC vs. WT-MSC). Data are presented as mean ± SEMs, $n = 6$.

**, $P < 0.01$; ***, $P < 0.001$(two-tailed Student's t test).

Figure S2. Transcriptomic analysis of brain tissues from the MCAO model mice after intracerebral administration of F3-Exo.

(A) The particle size distribution of exosomes measured by nanoparticle tracking analysis (NTA).

(B) Principal component analysis of brain samples from sham, vehicle, and F3-Exo groups.

(C) Volcano plot showing the MCAO DEGs and F3-Exo DEGs. Protein coding genes with top $\log_2$ (fold change) difference were labeled.

(D) Bar plots showing the representative GO terms enriched in MCAO DEGs and F3-Exo DEGs (p.adjust < 0.05). The length of the bar represents the $-\log_{10}$ (p.adjust) value.

(E) Heatmap showing the scaled expression levels of rescue up DEGs among sham, vehicle and F3-Exo groups. Color key from white to red or blue indicates the Z-score from low to high.

(F) Heatmaps showing the scaled expression levels of genes related to indicated pathways. Color key from white to red or blue indicates the Z-score from low to high.

Supplementary Table Legends

Supplementary Table S1. DEGs identified in MCAO mice between treatment with F3-Exo and vehicle, as well as DEGs between MCAO mice and sham-operated mice.

Supplementary Table S2. Primers used for genomic DNA PCR and RT-qPCR.

Supplementary Figure 1

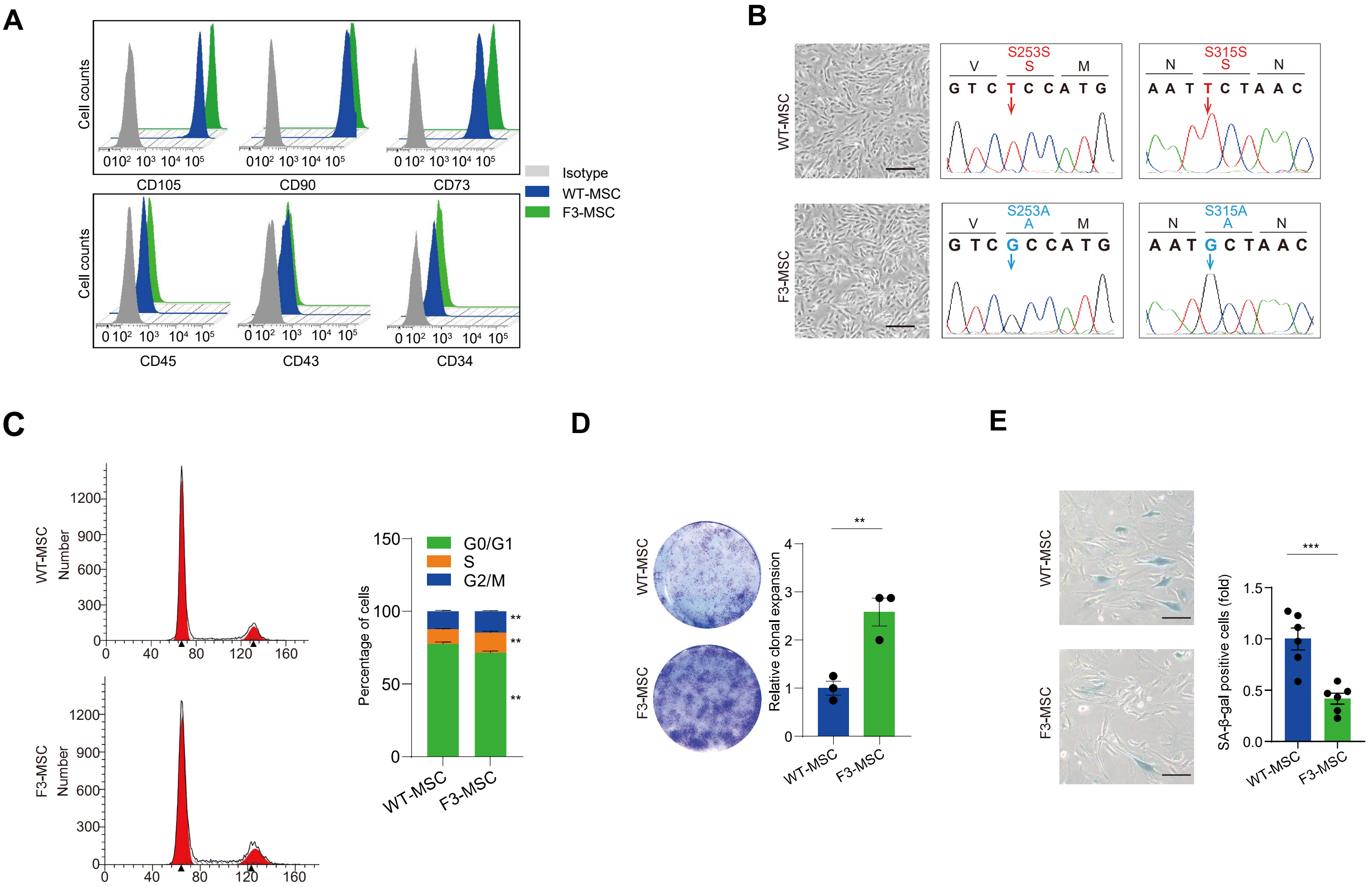

Supplementary Figure 2

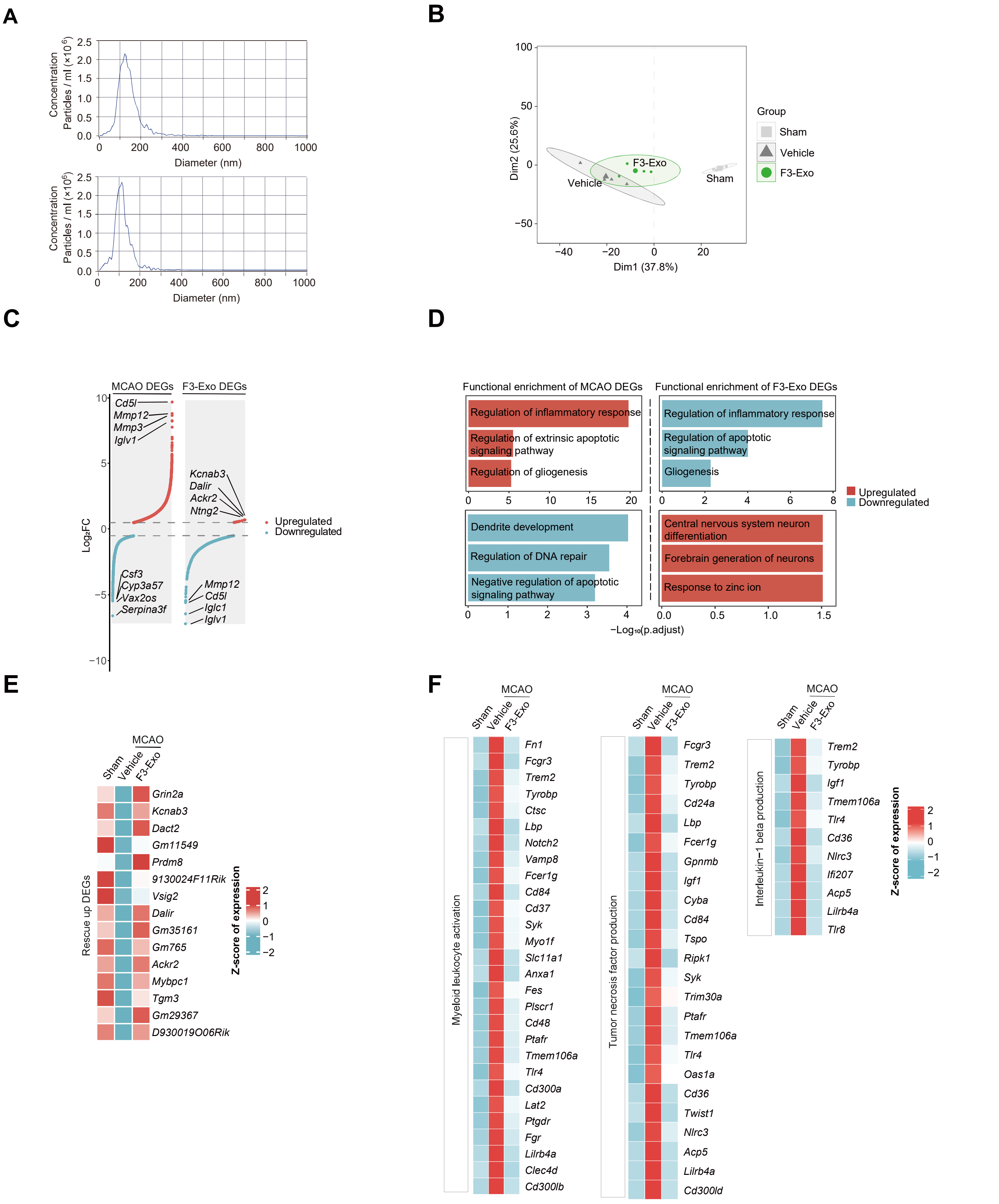

Supplement: pwaf004_suppl_Supplementary_Material [file pwaf004_suppl_supplementary_material.zip › Supplemental_materials.pdf]
